# INR Smooth: Interframe noise relation-based smooth video synthesis on diffusion models

**DOI:** 10.1371/journal.pone.0321193

**Published:** 2025-04-29

**Authors:** Cuihong Yu, Cheng Han, Chao Zhang

**Affiliations:** School of Computer Science and Technology, Changchun University of Science and Technology, Changchun, China; Universita degli Studi di Milano, ITALY

## Abstract

The text-to-video generation task can provide people with rich and diverse video content, but it also has some typical issues, such as content inconsistency between video frames or text alignment failure, which degrade the smoothness of video. And in the process of improving the video smoothing problems, the background texture and artistic expression are often lost because of the excessive smoothing. Based on the above problems, this paper proposes INR Smooth, a type of video smoothing strategy based on the relationship between interframe noise, which can improve the smoothness of most T2V generation tasks. Based on INR Smooth, two video smoothing editing methods are proposed in this paper. One is for T2V training models, based on the studied interframe noise relationship, noise constraints are carried out from the beginning and end of the video simultaneously, and video smoothing loss functions are constructed. The other is for T2V training-free models, this paper introduces DDIM Inversion additionally to ensure text alignment, so as to improve the smoothness. Through experimental comparison, it is found that the proposed methods can significantly improve text alignment, temporal consistency, and has outstanding performance in the smooth transition of real scenes and the portrayal of artistic styles. The proposed training-free method and zero-shot fine-tuning training method for video smoothing do not add additional computing resources. The source codes and video demos are available at https://github.com/Cuihong-Yu/INR-Smooth.

## Introduction

The purpose of text-to-video (T2V) generation is to generate realistic videos based on input text. As a new research hotspot of computer vision, text-to-video generation has been widely used in the fields of film and television production, games, entertainment short videos and so on [1]. The application potential of T2V is huge. The existing methods [2–7] mainly use large-scale diffusion models to achieve T2V tasks and achieve a series of stunning visual effects. Different from the research on image generation, video generation is greatly affected by the temporal consistency between frames and the spatial consistency of content features. If the consistency issue is not addressed effectively, it will result in flickering, incoherent action, and a reduction in video quality.

Tune-A-Video [8] simulates temporal consistency by fine-tuning the spatio-temporal attention mechanism, querying the relevant position of the current frame feature information in the previous frame. While Tune-A-Video generates significant text-to-video results with temporal consistency, unpleasant results may be generated when the input video contains multiple continuous objects. ControlVideo [9] employs the video keyframe attention mechanism for video consistency processing and introduces a temporal attention module to the diffusion model. A zero convolutional layer is added after each temporal attention module to preserve output before fine tuning. ControlVideo is affected by the guidance conditions, and there is still room for further improvement of video temporal consistency. Text2Video-Zero [10] introduces motion dynamic coding into potential coding and uses a new cross-frame attention mechanism to re-encode the self-attention mechanism of each frame to enforce temporal consistency. While the generated video has a smooth performance, the background content is relatively simple with no noticeable scene changes. In contrast to strategies that enhance the attention mechanism to improve video consistency, Stable Video Diffusion [11] adjusts the high-resolution text-to-video model into a frame interpolation model, uses the first and last frames as conditional frames, and feeds them to the UNet through the connection conditional mechanism, resulting in smooth videos at high frame rates. However, this frame interpolation method is limited by the length of the video.

The above advanced text-to-video generation models still face various problems that lead to the inconsistency of generated videos, so improving the temporal consistency of generated videos is the main challenge faced by text-to-video. In order to maintain the creativity of the original model and further improve video consistency, consistent video editing methods can be further explored. Therefore, this paper studies consistent video editing based on the noise relationship between video frames. Relatively few studies have investigated the inter-frame noise of text-to-video generation. PYoCo [12] investigated the correlation of noises corresponding to each frame of a video and thus developed mixed and progressive noise priors to guide the video training process, resulting in the consistency of sequential video frame generation. Smooth Video [13] introduces a loss term to noise predictions for adjacent video frames to enhance the edited object’s smoothness. However, the over-smoothing limits the alignment between the generated and source videos.

**Fig 1:**
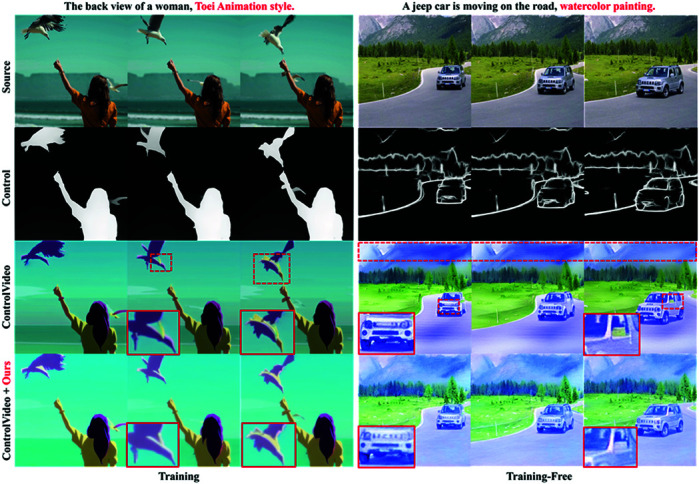
INR Smooth: A video smoothing strategy based on the interframe noise relation. Based on INR Smooth, two video smoothing editing methods are proposed, one is for T2V training models, the other is for T2V training-free models. As shown in Fig 1, after employing the proposed methods, the body structure of the bird is repaired, the front structure of the car is aligned with other frames, and the color of the rearview mirror is repaired.

Based on the challenges of improving the consistency of text-to-video, this paper proposes INR Smooth, a video smoothing strategy for linking noise relation between different frames in video, which improves the inconsistency of existing models, enhances scene transitions, reduces over-smoothing, and maintains alignment with the source video. It is applicable to video generation and editing models, and compatible with the current personalized or conditional T2V generation tasks. Fig 1 illustrates the remarkable results of INR Smooth in T2V video smoothing application. By extensive qualitative and quantitative comparisons with state-of-the-art baselines, we demonstrate the superiority of the proposed methods. The main contributions of this work are as follows:

- Based on the diffusion models, the relation between the noise of each frame in the T2V model is studied, and a video smoothing strategy based on interframe noise relation is proposed.
- For T2V training models, this paper constructs the video smoothing loss based on the interframe noise relation, so that the video smoothing loss and original loss affect the quality of the generated video simultaneously.
- For T2V training-free models, we propose an improved version of DDIMs (Denoising Diffusion Implicit Models) inversion, which is suited for zero-shot video editing. By analyzing the correlation between inversion noise and random noise, inversion noise is estimated from the beginning and end of the video simultaneously. Finally, the weight of inversion noise is controlled to maintain the interframe consistency of generated video.

## Related work

### Text-to-video generation

The large-scale text-to-image (T2I) diffusion models has achieved unprecedented success, so researchers focus on applying the diffusion models to the field of video generation, and have achieved a series of remarkable research results. While many research [1,4,6,11] bring visual impact to people, but super computing resources make it difficult for individual researchers or small research teams, so researchers try to study how to better achieve text-to-video generation on the basis of lightweight resource utilization. For example, many T2V studies based on Zero-Shot, One-Shot, Few Shot and Training-Free have achieved better generation effects. This paper will also focus on lightweight resource utilization research for smooth editing.

Many major T2V generation models use the T2I model’s merging time module to generate video. Video Diffusion Models [14] extend the basic U-Net architecture of 2D image modeling to 3D space-time by factorizing the spatiotemporal attention module, and realize use the standard equation of diffusion model, which can obtain an effective process of video data generation model. Sora [15] explores large-scale training of generative models on video data, and leverages a transformer architecture that operates on spacetime patches of video and image latent codes, Sora demonstrates the feasibility of scaled video generation models. Imagen Video [16] improves video diffusion models by cascading them and using V-parametric decision making. Make-A-Video [17] begins with learning content description and move features and then extends a diffusion models-based T2I model [18] to T2V using a spatiotemporally factorized diffusion model. Lumiere [19] introduces a Space-Time U-Net architecture based on the pre-trained T2I U-Net to generate the entire temporal duration of the video, which synthefies state-of-the-art diverse and coherent videos. Follow Your Pose [20] also applies the image generation diffusion model to the video generation process, proposing a pose-guided text-to-video generation model to create a character video generation model with editable text and pose management. In addition to the guiding conditions such as pose, edge, and semantic segmentation, T2V tasks are evolving toward multi-conditional guidance. Therefore, in the face of extensive developing T2V generating tasks, research on video smoothing methods will be conducted throughout and is very important.

### Text-to-video editing

The video editing based on diffusion models is often guided by text, so as to change the content, style and background layout of the generated video. Dreamix [21] proposed a text-based general appearance and action editing method for realistic scene videos, which achieved good video editing effects, but the model implementation process required relatively high computing resources. Patrick Esser [22] et al. proposed a structure and content perception model, which can modify video through sample images or text guidance, and demonstrated for the first time the control of time consistency by the joint training of image and video data. The experimental results are impressive, but the implementation process requires large-scale text image data resources. FateZero [23] captures the intermediate attention graph in the inversion process to retain the structure and motion information, fuse self-attentions with a blending mask obtained by cross-attention features from the source prompt, and changes the self-attention in the UNet to spatio-temporal attention, so as to realize the change of the video foreground content and the editing of the video style. Rerender A Video [24] presents a novel video-to-video translation framework, which translates the source video into the target video based on the style description of the text. It can be widely used in video style transfer. TokenFlow [25] finds that enhancing the consistency of internal diffusion features between frames can generate videos with temporal consistency during video editing. Video-P2P [26] achieve video editing by introducing cross-attention control and optimizing shared unconditional embedding of video inversion, as well as using different guides for source and editing prompts, and merging their attention. Slicedit [27] runs DDPM sampling using the extracted source video noise space, while injecting the guided conditional attention maps at specific timesteps to achieve video editing. FLATTEN [28] extends the T2I diffusion model and incorporates flow-guided attention into the DDIM sampling and inversion process, resulting in consistent video editing results. With the vigorous development of T2V tasks, T2V editing tasks do not require huge computing resources for the generation process, and can be flexible in the output video style, so T2V editing tasks will also be widely developed.

### Latent diffusion models

Diffusion models have recently emerged as new leaders in deep generation models, owing to their high-quality generation effects in image generation tasks. They have excellent performance in many application fields, such as computer vision, NLP (Natural Language Processing), multimodal modeling, medical image reconstruction and so on [29]. Since the original diffusion models still has some shortcomings, such as slow sampling speed, poor maximization likelihood and weak data generalization ability, many optimization studies based on diffusion models have been conducted based on the above problems [30–36].

In this paper, video smoothing is studied on the basis of latent diffusion models [18]. Compared with other space compression methods, latent diffusion models propose a method to perform the diffusion process in latent space, which can reduce the computational complexity and achieve better image generation. Instead of training the model on pixel space, latent diffusion models process the original images through self-coding models, retaining only some features of the important foundation, thus greatly reducing the complexity of the training and sampling phases. latent diffusion models mainly consist of a self-coding model (including an encoder and a decoder) and diffusion operations of the latent space.

Specifically, given an image $x\in {\mathbb{R}}^{H\times W\times 3}$, *H* is the height, *W* is the width, and 3 is the number of color channels (R, G, B). First use an encoder *∈* encode the image into the latent space z=∈ (x), where $z\in {\mathbb{R}}^{h\times w\times c}$, then use a decoder to reconstruct the image from the latent space $\tilde{x}=D\left(z\right)=D\left(\in \left(x\right)\right)$, the downsampling factor is *f* = *H* ∕ *h* = *W* ∕ *w*. In the latent space, noise prediction network $\varepsilon_{\theta }\left(z_{t},t\right)$ need to be trained to implement the denoising process, $z_{t}$ is a noisy sample of $z_{0}$ at timestep *t*, *t* = 1 , . . . , *T*. In the latent diffusion models, a self-coding model is introduced, so the $z_{t}$ obtained by the encoder can be used to make the model learn in the latent space during training, expressed as:


$$\begin{array}{ccc} L_{LDM}={𝔼}_{\in (x),\varepsilon ∼\;N(0,1),t}\;[||\varepsilon -\varepsilon_{\theta }(z_{t},t)|{|}_{2}^{2}] & & \end{array}$$ (1)

where noise *ε* is added to $z_{0}$ according to step *t* to obtain $z_{t}$. For the conditional guided image generation process, LDM introduces a domain-specific encoder $\tau_{\theta }$ for pre-processing condition *y*, where learning can be expressed as:


$$\begin{array}{ccc} L_{LDM}={𝔼}_{\in (x),y,\varepsilon ∼\;N(0,1),t}\;[||\varepsilon -\varepsilon_{\theta }(z_{t},t,\tau_{\theta }(y))|{|}_{2}^{2}] & & \end{array}$$ (2)

### Denoising diffusion implicit models (DDIMs)

The denoising diffusion implicit models (DDIMs) [30] are more efficient iterative implicit probability models that build on the denoising diffusion probability models (DDPMs) [37] to enhance sampling speed even further. In DDPMs, the generative process is defined as the reverse of a particular Markovian diffusion process, so the generation steps are continuous and non-skippable. However, DDIMs break the Markovian process through mathematical reasoning and realize multi-step sampling, which improves sampling speed. The non-Markovian process is derived as follows:

Analyze the inference diffusion process indexed by real vectors $\sigma \in {\mathbb{R}}_{\geq 0}^{<T}$:


$$\begin{array}{ccc} q_{\sigma }(x_{1:T}|x_{0}):=q_{\sigma }(x_{T}|x_{0})\overset{T}{\underset{t=2}{\prod }}q_{\sigma }(x_{t-1}|x_{t},x_{0}) & & \end{array}$$ (3)

where $x_{t}$ is a latent variable, timestep *t* = 1 , . . . , *T*, $q(x_{0})$ is a data distribution, when *t*>1 and diffusion hyperparameter $\alpha_{T}$ sufficiently close to 0, $q_{\sigma }\left(x_{T}|x_{0}\right)=N\left(\sqrt{\alpha_{T}}x_{0},\left(1-\alpha_{T}\right)I\right)$, therefore, the following equation can be obtained:


$$\begin{array}{ccc} q_{\sigma }(x_{t-1}|x_{t},x_{0})=N(\sqrt{\alpha_{t-1}}x_{0}+\sqrt{1-\alpha_{t-1}-\sigma_{t}^{2}}\cdot \frac{x_{t}-\sqrt{\alpha_{t}}x_{0}}{\sqrt{1-\alpha_{t}}},\sigma_{t}^{2}I) & & \end{array}$$ (4)

According to Bayes’ rule, the following forward process can be derived:


$$\begin{array}{ccc} q_{\sigma }(x_{t}|x_{t-1},x_{0})=\frac{q_{\sigma }(x_{t-1}|x_{t},x_{0})q_{\sigma }(x_{t}|x_{0})}{q_{\sigma }(x_{t-1}|x_{0})} & & \end{array}$$ (5)

where the forward process is no longer a Markovian process, because every $x_{t}$ can depend on $x_{t-1}$, $x_{0}$.

The generation process is as follows:

Define a trainable generation process $p_{\theta }\left(x_{0}:T\right)$, where each $p_{\theta }^{\left(t\right)}(x_{t-1}|x_{t})$ leverages knowledge of $q_{\sigma }(x_{t-1}|x_{t},x_{0})$. Given a noise observation $x_{t}$, $\varepsilon_{t}∼N\left(0,I\right)$, model $\varepsilon_{\theta }^{\left(t\right)}\left(x_{t}\right)$ predict $\varepsilon_{t}$ from $x_{t}$, which is a prediction of $x_{0}$ given $x_{t}$:


$$\begin{array}{ccc} f_{\theta }^{\left(t\right)}(x_{t}):=\frac{\left(x_{t}-\sqrt{1-\alpha_{t}}\cdot \varepsilon_{\theta }^{\left(t\right)}(x_{t})\right)}{\sqrt{\alpha_{t}}} & & \end{array}$$ (6)

The generation process can be defined as follows:


$$\begin{array}{ccc} p_{\theta }^{\left(t\right)}(x_{t-1}|x_{t})=\{\begin{array}{c} N(f_{\theta }^{\left(1\right)}(x_{1}),\sigma_{1}^{2}I),if\;t=1\\ q_{\sigma }(x_{t-1}|x_{t},f_{\theta }^{\left(t\right)}(x_{t})),otherwise \end{array} & & \end{array}$$ (7)

According to the generation process, the implicit model equation of denoising diffusion is further obtained as follows:


$$\begin{array}{ccc} x_{t-1}=\sqrt{\alpha_{t-1}}(\frac{x_{t}-\sqrt{1-\alpha_{t}}\varepsilon_{\theta }^{\left(t\right)}(x_{t})}{\sqrt{\alpha_{t}}})+\sqrt{1-\alpha_{t-1}-\sigma_{t}^{2}}\cdot \varepsilon_{\theta }^{\left(t\right)}(x_{t})+\sigma_{t}\varepsilon_{t} & & \end{array}$$ (8)

By setting $\sigma_{t}$ to 0, the sampling process is deterministic, the latent variables are consistent, and the sampling steps are reduced. The following equation is obtained:


$$\begin{array}{ccc} x_{t-1}=\sqrt{\alpha_{t-1}}(\frac{x_{t}-\sqrt{1-\alpha_{t}}\varepsilon_{\theta }^{\left(t\right)}(x_{t})}{\sqrt{\alpha_{t}}})+\sqrt{1-\alpha_{t-1}}\cdot \varepsilon_{\theta }^{\left(t\right)}(x_{t}) & & \end{array}$$ (9)

## Method

**Fig 2:**
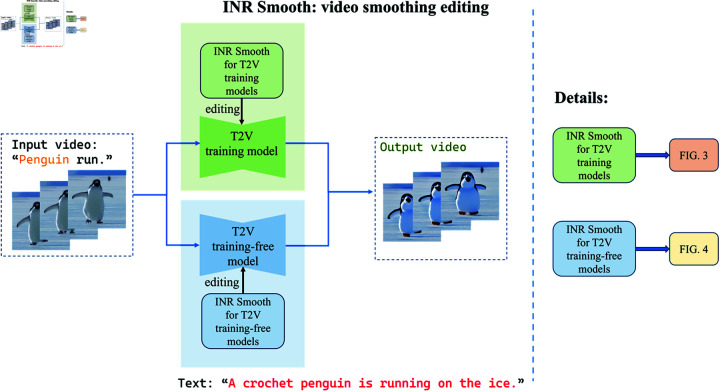
Framework. Based on the proposed INR Smooth strategy, two video smoothing editing methods are proposed, one for T2V training models (green) and one for T2V training-free models (blue).

In this section, the noise relation between different frames during T2V sampling will be analyzed and studied, and then the interframe noise relation model will be constructed to improve the consistency between different frames. For both training and training-free T2V methods, specific smoothing editing methods are proposed based on interframe noise relation model. The proposed methods can meet the smoothing requirements of most T2V studies and have strong generalization ability. The framework is illustrated in Fig 2.

### Research on the interframe noise relation

The training and sampling process of the diffusion models are carried out around the noise. As the core content of the diffusion models, noise brings infinite possibilities to researchers. For the traditional T2I model, the process of add noise or denoising is only for static images, but the video generated by the T2V model is composed of a series of static images, so the process of T2V generation will increase resources according to the length and resolution of the video. We often consider the noise relation of each step in the process of adding noise and denoising for T2I, so what is the relation between the noise of different frames in T2V, and how does it affect the consistency of the video? At present, relatively few studies [12,13] have been carried out on the noise relationship between video frames, and our research work is carried out in this aspect.

For a single frame image, in the forward process, noise is progressively added to the observation $x_{0}$, and $x_{t}$ is obtained after t steps, for any step t, $x_{t}$ can be expressed as a linear combination of $x_{0}$ and random noise variable $\varepsilon_{t}$:


$$\begin{array}{ccc} x_{t}=\sqrt{{\bar{\alpha }}_{t}}x_{0}+\sqrt{1-{\bar{\alpha }}_{t}}\varepsilon_{t} & & \end{array}$$ (10)

In the sampling process, after progressively denoise from $x_{t}$, the observation ${\hat{x}}_{0}$ is finally obtained, then $x_{t}$ can also be expressed as a linear combination of ${\hat{x}}_{0}$ and estimated noise variable ${\hat{\varepsilon }}_{t}$:


$$\begin{array}{ccc} x_{t}=\sqrt{{\bar{\alpha }}_{t}}{\hat{x}}_{0}+\sqrt{1-{\bar{\alpha }}_{t}}{\hat{\varepsilon }}_{t} & & \end{array}$$ (11)

Discuss the relation between the noise of different frames in T2V. In the forward process, $x_{0}^{n-1}$, $x_{0}^{n}$ and $x_{0}^{n+1}$ represent the original observations corresponding to any adjacent three frames, *n* is the frame index, after t steps, random noise variables $\varepsilon_{t}^{n-1}$, $\varepsilon_{t}^{n}$, $\varepsilon_{t}^{n+1}$ are added to the original observations, then $x_{t}^{n-1}$, $x_{t}^{n}$ and $x_{t}^{n+1}$ are obtained.

In the sampling process, after progressively denoise from the adjacent three frames, ${\hat{x}}_{0}^{n-1}$, ${\hat{x}}_{0}^{n}$ and ${\hat{x}}_{0}^{n+1}$ are obtained; ${\hat{\varepsilon }}_{t}^{n-1}$, ${\hat{\varepsilon }}_{t}^{n}$, ${\hat{\varepsilon }}_{t}^{n+1}$ respectively represent the estimated noise variable of the three frames during the denoising process. The diffusion hyperparameter ${\bar{\alpha }}_{t}$ values for the adjacent three frames in the same step are equal, so we can express any adjacent three frames as follows:


$$\begin{array}{ccc} \sqrt{{\bar{\alpha }}_{t}}x_{0}^{n+1}+\sqrt{1-{\bar{\alpha }}_{t}}\varepsilon_{t}^{n+1}=x_{t}^{n+1}=\sqrt{{\bar{\alpha }}_{t}}{\hat{x}}_{0}^{n+1}+\sqrt{1-{\bar{\alpha }}_{t}}{\hat{\varepsilon }}_{t}^{n+1} & & \end{array}$$ (12)

$$\begin{array}{ccc} \sqrt{{\bar{\alpha }}_{t}}x_{0}^{n}+\sqrt{1-{\bar{\alpha }}_{t}}\varepsilon_{t}^{n}=x_{t}^{n}=\sqrt{{\bar{\alpha }}_{t}}{\hat{x}}_{0}^{n}+\sqrt{1-{\bar{\alpha }}_{t}}{\hat{\varepsilon }}_{t}^{n} & & \end{array}$$ (13)

$$\begin{array}{ccc} \sqrt{{\bar{\alpha }}_{t}}x_{0}^{n-1}+\sqrt{1-{\bar{\alpha }}_{t}}\varepsilon_{t}^{n-1}=x_{t}^{n-1}=\sqrt{{\bar{\alpha }}_{t}}{\hat{x}}_{0}^{n-1}+\sqrt{1-{\bar{\alpha }}_{t}}{\hat{\varepsilon }}_{t}^{n-1} & & \end{array}$$ (14)

For forward and sampling processes, the ultimate prediction objective is to reach $x_{0}\approx {\hat{x}}_{0}$ or $\varepsilon_{t}\approx {\hat{\varepsilon }}_{t}$ and construct loss from there, so according to the prediction objective, we can construct the following equation:


$$\begin{array}{ccc} \varepsilon_{t}^{n+1}\approx {\hat{\varepsilon }}_{t}^{n+1},\varepsilon_{t}^{n}\approx {\hat{\varepsilon }}_{t}^{n} & & \end{array}$$ (15)

$$\begin{array}{ccc} \varepsilon_{t}^{n-1}\approx {\hat{\varepsilon }}_{t}^{n-1},\varepsilon_{t}^{n}\approx {\hat{\varepsilon }}_{t}^{n} & & \end{array}$$ (16)

Transform the two above equations as follows:


$$\begin{array}{ccc} \varepsilon_{t}^{n+1}{\hat{\varepsilon }}_{t}^{n}\approx {\hat{\varepsilon }}_{t}^{n+1}\varepsilon_{t}^{n} & & \end{array}$$ (17)

$$\begin{array}{ccc} \varepsilon_{t}^{n-1}{\hat{\varepsilon }}_{t}^{n}\approx {\hat{\varepsilon }}_{t}^{n-1}\varepsilon_{t}^{n} & & \end{array}$$ (18)

As shown in the above equations, for any adjacent three frames, when the steps are same, the increased noise variable and the estimated noise variable have a certain constraint relation, which will certainly affect the consistency of each frame.

### INR Smooth: Video smoothing editing method for T2V training models

**Fig 3:**
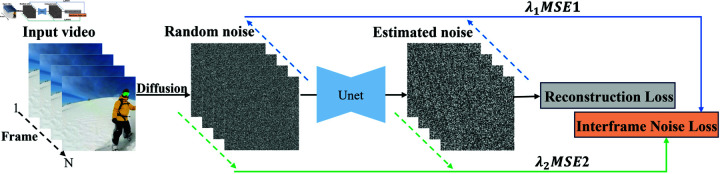
INR Smooth: Video smoothing editing framework for T2V training models. The green arrow indicates the direction from the first frame to the last frame to build the interframe noise constraint, and the blue arrow indicates the opposite process.

In order to improve the alignment of text and video and the consistency of different frames, the above results of the interframe noise relation can be applied to the T2V training models. Fig 3 depicts the proposed video smoothing editing framework for T2V training models.

As shown in Fig 3, construct interframe noise constraints from both directions simultaneously, then obtain the mean square error (MSE) of the interframe noise variables:


$$\begin{array}{ccc} MSE(1)=\frac{1}{N-1}\sum_{n=1}^{N-1}{\left(\varepsilon_{t}^{n+1}{\hat{\varepsilon }}_{t}^{n}-{\hat{\varepsilon }}_{t}^{n+1}\varepsilon_{t}^{n}\right)}^{2} & & \end{array}$$ (19)

$$\begin{array}{ccc} MSE(2)=\frac{1}{N-1}\sum_{n=2}^{N}{\left(\varepsilon_{t}^{n-1}{\hat{\varepsilon }}_{t}^{n}-{\hat{\varepsilon }}_{t}^{n-1}\varepsilon_{t}^{n}\right)}^{2} & & \end{array}$$ (20)

where *n* indexes the corresponding video frame, *N* is the total number of frames of the video, *MSE*(1) represents the interframe noise constraint from the last frame to the first, *MSE*(2) represents the interframe noise constraint from the first frame to the last. The video smoothing loss function of the training model is constructed as follows:


$$\begin{array}{ccc} L_{smooth}=\lambda_{1}MSE(1)+\lambda_{2}MSE(2) & & \end{array}$$ (21)

where $\lambda_{1}$ and $\lambda_{2}$ are the weights of the constraint relation between frames in two directions respectively, and the values of $\lambda_{1}$ and $\lambda_{2}$ can be set to 0.5 respectively, which can be adjusted appropriately according to the characteristics of experimental objects during the experiment. Thus, the overall loss function of the T2V training model can be obtained as follows:


$$\begin{array}{ccc} Loss=L_{org}+\lambda L_{smooth} & & \end{array}$$ (22)

where *λ* is the weight of video smoothing loss, and $L_{org}$ is the original loss function of the diffusion models. The pseudocode of the video smoothing editing method for the T2V training models is shown in Algorithm 1.

### INR Smooth: Video smoothing editing method for T2V training-free models

On the basis of large-scale diffusion models, more and more researchers begin to explore lightweight optimization research that can be achieved only through model fine-tuning without adding additional computing resources and training. The training-free T2V generating process uses fewer computing resources and produces results faster. However, due to the lack of training and learning of the study samples, it is often difficult to control the smooth performance of the generated video. To address such research challenges, DDIM Inversion is introduced based on the above interframe noise relation, and control the inversion noise to improve the smoothness of the generated video.

The purpose of DDIM Inversion is to reverse the sampling process, resulting in a noisy implicit representation $x_{t}$ that serves as the starting for the sampling process, and the generated image will be close to the source image. The whole process can be modeled as the process of adding random noise $\varepsilon_{t}$ to $x_{0}$ gets $x_{t}$, then using $x_{t}$ as the starting point for DDIM sampling to generate ${\hat{x}}_{t-1}$ and predict inversion noise (represented by $r{\hat{\varepsilon }}_{t}$), and then gradually sampling to get the final generated image. From , we can generate a sample $x_{t}$ from a sample $x_{t-1}$ via:


$$\begin{array}{ccc} x_{t}=\frac{\sqrt{{\bar{\alpha }}_{t}}}{\sqrt{{\bar{\alpha }}_{t}-1}}(x_{t-1}-\sqrt{1-{\bar{\alpha }}_{t-1}}\varepsilon_{t})+\sqrt{1-{\bar{\alpha }}_{t}}\varepsilon_{t} & & \end{array}$$ (23)

**Algorithm 1: Video smoothing editing method for T2V training models.**

**Input:**   $x_{0}$

**Output:**   ${\hat{x}}_{0}$

1:   **Hyperparameters:** $\lambda_{1}$, $\lambda_{2}$

2:   Diffusion process

3:   **for** *t* = 1 , 2 , . . . , *T* **do**

4:    $x_{t}=\sqrt{{\bar{\alpha }}_{t}}x_{0}+\sqrt{1-{\bar{\alpha }}_{t}}\varepsilon_{t}$

5:   **end** **for**

6:   Denoising process

7:   **for** *t* = *T* , ( *T* − 1 ) , . . . , 1 **do**

8:    $x_{t}=\sqrt{{\bar{\alpha }}_{t}}{\hat{x}}_{0}+\sqrt{1-{\bar{\alpha }}_{t}}{\hat{\varepsilon }}_{t}$

9:   **end** **for**

10:   Deriving the noise relationship between video frames

11:   **from**

12:   $\sqrt{{\bar{\alpha }}_{t}}x_{0}^{n+1}+\sqrt{1-{\bar{\alpha }}_{t}}\varepsilon_{t}^{n+1}=x_{t}^{n+1}=\sqrt{{\bar{\alpha }}_{t}}{\hat{x}}_{0}^{n+1}+\sqrt{1-{\bar{\alpha }}_{t}}{\hat{\varepsilon }}_{t}^{n+1}$

13:   $\sqrt{{\bar{\alpha }}_{t}}x_{0}^{n}+\sqrt{1-{\bar{\alpha }}_{t}}\varepsilon_{t}^{n}=x_{t}^{n}=\sqrt{{\bar{\alpha }}_{t}}{\hat{x}}_{0}^{n}+\sqrt{1-{\bar{\alpha }}_{t}}{\hat{\varepsilon }}_{t}^{n}$

14:   $\sqrt{{\bar{\alpha }}_{t}}x_{0}^{n-1}+\sqrt{1-{\bar{\alpha }}_{t}}\varepsilon_{t}^{n-1}=x_{t}^{n-1}=\sqrt{{\bar{\alpha }}_{t}}{\hat{x}}_{0}^{n-1}+\sqrt{1-{\bar{\alpha }}_{t}}{\hat{\varepsilon }}_{t}^{n-1}$

15:   **to**

16:   $\varepsilon_{t}^{n+1}{\hat{\varepsilon }}_{t}^{n}\approx {\hat{\varepsilon }}_{t}^{n+1}\varepsilon_{t}^{n}$

17:   $\varepsilon_{t}^{n-1}{\hat{\varepsilon }}_{t}^{n}\approx {\hat{\varepsilon }}_{t}^{n-1}\varepsilon_{t}^{n}$

18:   Constructing smoothing loss

19:   $MSE(1)=\frac{1}{N-1}\sum_{n=1}^{N-1}{\left(\varepsilon_{t}^{n+1}{\hat{\varepsilon }}_{t}^{n}-{\hat{\varepsilon }}_{t}^{n+1}\varepsilon_{t}^{n}\right)}^{2}$

20:   $MSE(2)=\frac{1}{N-1}\sum_{n=2}^{N}{\left(\varepsilon_{t}^{n-1}{\hat{\varepsilon }}_{t}^{n}-{\hat{\varepsilon }}_{t}^{n-1}\varepsilon_{t}^{n}\right)}^{2}$

21:   $L_{smooth}=\lambda_{1}MSE(1)+\lambda_{2}MSE(2)$

22:   **return** ${\hat{x}}_{0}$

**Fig 4:**
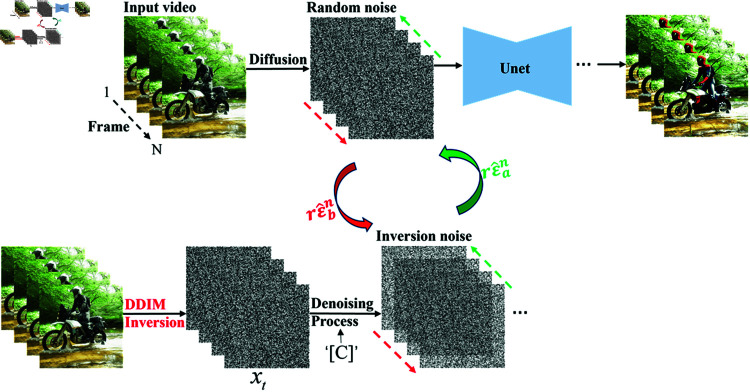
INR Smooth: Video smoothing editing framework for training-free models. Different from the smoothing editing method of the training models, inversion noise is used to replace the estimated noise for the training-free models.

According to the DDIM Inversion process and the interframe noise relation studied in this paper, a video smoothing editing framework for training-free models is constructed, as shown in Fig 4.

As shown, random noise is added in the diffusion forward process, and inversion noise is removed by the DDIM Inversion sampling process, $r{\hat{\varepsilon }}_{a}^{n}$ represents the inversion noise from the last frame to the first, $r{\hat{\varepsilon }}_{b}^{n}$ represents the inversion noise from the first frame to the last, *a* and *b* represent the step, *n* indexes the corresponding video frame. The noise constraint from both ends of the video is conducive to improving the consistency between video frames. According to Eqs 17 and 18 and DDIM Inversion process, $r{\hat{\varepsilon }}_{a}^{n}$ and $r{\hat{\varepsilon }}_{b}^{n}$ can be constructed as follows:


$$\begin{array}{ccc} r{\hat{\varepsilon }}_{a}^{n}={\left(\varepsilon_{a}^{n+1}\right)}^{-1}\varepsilon_{a}^{n}r{\hat{\varepsilon }}_{a}^{n+1} & & \end{array}$$ (24)

$$\begin{array}{ccc} r{\hat{\varepsilon }}_{b}^{n}={\left(\varepsilon_{b}^{n-1}\right)}^{-1}\varepsilon_{b}^{n}r{\hat{\varepsilon }}_{b}^{n-1} & & \end{array}$$ (25)

For training-free models, inversion noise is used to replace the estimated noise in the method for training models. Inversion noise $r{\hat{\varepsilon }}_{a}^{n}$ has a value starting from the second-to-last frame of the video, inversion noise $r{\hat{\varepsilon }}_{b}^{n}$ has a value starting from the second frame of the video, they both depend on the added random noise and the removed inversion noise of the adjacent frame. Weighting the inversion noise in both directions to obtain the inversion noise $r{\hat{\varepsilon }}_{t}^{n}$ of the NTH frame:


$$\begin{array}{ccc} r{\hat{\varepsilon }}_{t}^{n}=\left(1-\lambda_{r}\right){\hat{\varepsilon }}_{t}^{n}+\lambda_{r}\left(\frac{1}{2}r{\hat{\varepsilon }}_{a}^{n}+\frac{1}{2}r{\hat{\varepsilon }}_{b}^{n}\right) & & \end{array}$$ (26)

where $\lambda_{r}$ is inverse noise weight, by adjusting the inverse noise weight, the interframe consistency and text alignment of the video generated by the training-free T2V models can be improved. The pseudocode of the video smoothing editing method for the T2V training-free models is shown in Algorithm 2.

**Algorithm 2.** Video smoothing editing method for T2V training-free models.

**Input:**   $x_{0}$

**Output:**   ${\hat{x}}_{0}$

1:   **Hyperparameters:** $\lambda_{r}$

2:   DDIM for inversion latents

3:   **for** *t* = 1 , 2 , . . . , *T* **do**

4:    $x_{t}=\frac{\sqrt{{\bar{\alpha }}_{t}}}{\sqrt{{\bar{\alpha }}_{t}-1}}(x_{t-1}-\sqrt{1-{\bar{\alpha }}_{t-1}}\varepsilon_{t})+\sqrt{1-{\bar{\alpha }}_{t}}\varepsilon_{t}$

5:   **end** **for**

6:   Deriving the noise relationship between frames by inversion noise and random noise

7:   $r{\hat{\varepsilon }}_{a}^{n}={\left(\varepsilon_{a}^{n+1}\right)}^{-1}\varepsilon_{a}^{n}r{\hat{\varepsilon }}_{a}^{n+1}$

8:   $r{\hat{\varepsilon }}_{b}^{n}={\left(\varepsilon_{b}^{n-1}\right)}^{-1}\varepsilon_{b}^{n}r{\hat{\varepsilon }}_{b}^{n-1}$

9:   Constructing the inversion noise

10:   $r{\hat{\varepsilon }}_{t}^{n}=\left(1-\lambda_{r}\right){\hat{\varepsilon }}_{t}^{n}+\lambda_{r}\left(\frac{1}{2}r{\hat{\varepsilon }}_{a}^{n}+\frac{1}{2}r{\hat{\varepsilon }}_{b}^{n}\right)$

11:   **return** ${\hat{x}}_{0}$

## Experiments

### Implementation details

The proposed two smoothing editing methods can be applied to T2V baselines which require training or inversion-based baselines which are training-free, respectively. The implementation is based on each baseline’s official codes, original data frame rate, frame number, learning rate, batch size and resolution requirements. The comparison experiments with baselines are representative. In the inference phase, we use DDIM sampler with classifier-free guidance. As the internal parameters of different baselines are obviously different, in order to adapt to the optimization of different baselines, it is necessary to adjust the weights according to specific baselines. So the smoothing loss weight *λ* and inverse noise weight $\lambda_{r}$ can be set according to the concrete experiment. The number of sampling steps in the experiment is 50. All experiments are conducted on an NVIDIA RTX 4090 GPU.

### Dataset and metrics

This study uses official implementation datasets of each baseline for smoothing tests, which mainly include the DAVIS dataset [38]. We evaluate the quality of the generated video from two aspects: text alignment and frame consistency. According to the previous research [8,9,22,23,26], we select CLIP (T) (text alignment evaluation), CLIP (F) (frame consistency evaluation), and Optical flow [39] as the corresponding evaluation metrics. CLIP (T) refers to the average cosine similarity of text and image embedded across all frames of the generated video. CLIP (F) refers to the average cosine similarity between all consecutive frame pairs of the generated video. Optical flow computes the motion vector of each pixel between two frames using image information from succeeding frames. For the smoothing optimization effect of the baselines, the source videos provided by the official implementation process will be used as the main evaluation and comparison.

### Baselines

Based on the generated data from the state-of-the-art baselines: Tune-A-Video [8], ControlVideo [9], FateZero [23] and Video-P2P [26], this paper carries out smoothing editing experiments. Smoothing editing methods include Smooth Video [13] and the proposed methods. At the same time, two advanced methods Slicedit [27] and STEM [40] are selected for extended comparison. Among them, Smooth Video [13] applies a video frame noise prediction method, which is in contrast with the proposed methods. Slicedit [27] and STEM [40] apply DDPM inversion and Spatial-Temporal Expectation-Maximization inversion, respectively, in contrast to the DDIM inversion applied in this paper.

### Smoothing results based on training baselines

**Comparison with Tune-A-Video.** The experiments are based on the Stable Diffusion 1.4, with the guidance_scale set at 12.5. The experiments compare the baseline methods Tune-A-Video [8], Slicedit [27], and STEM [40], the smoothing editing method Smooth Video [13], and the proposed video smoothing editing method (Ours(T)) which is based on the training models. The smoothing loss weight of Ours(T) is set to 2.0.

**Fig 5:**
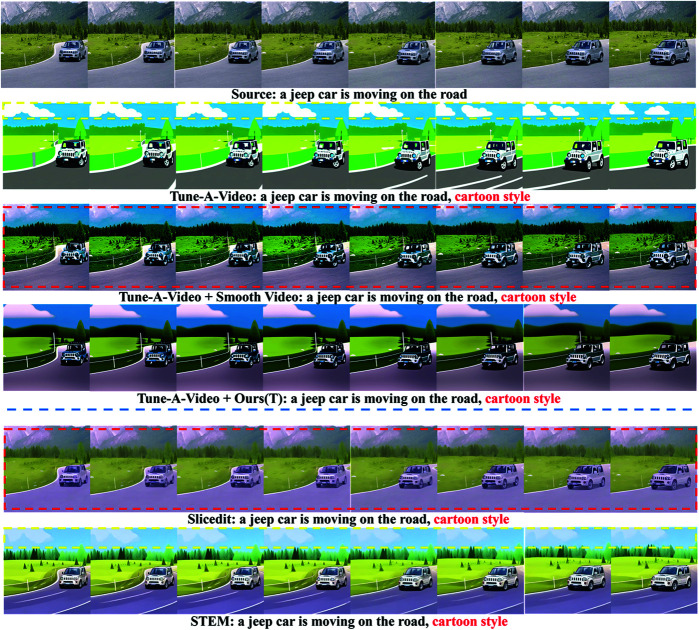
Comparison of smoothing editing results of the cartoon style video generated by Tune-A-Video.

As shown in Fig 5, each row in the figure is the middle 8 frames with obvious changes selected from the generated video. As shown, the video generated by Tune-A-Video can maintain the semantic alignment with the text, but there are still obvious flickers between frames (as shown in the yellow box of Tune-A-Video). With the introduction of Smooth Video, the smoothness of Tune-A-Video is improved, but the video results do not present a cartoon style and thus are not aligned with the text semantics. After introducing the proposed smoothing method (Ours(T)), the generated video exhibits better frame consistency, while the generated video has better quality and obvious cartoon style. The generated results of Slicedit do not present cartoon style and do not achieve text alignment. The STEM generation effect is remarkable, the text is well aligned, but there are slightly unsmooth details (as shown in the yellow box of STEM).

**Fig 6:**
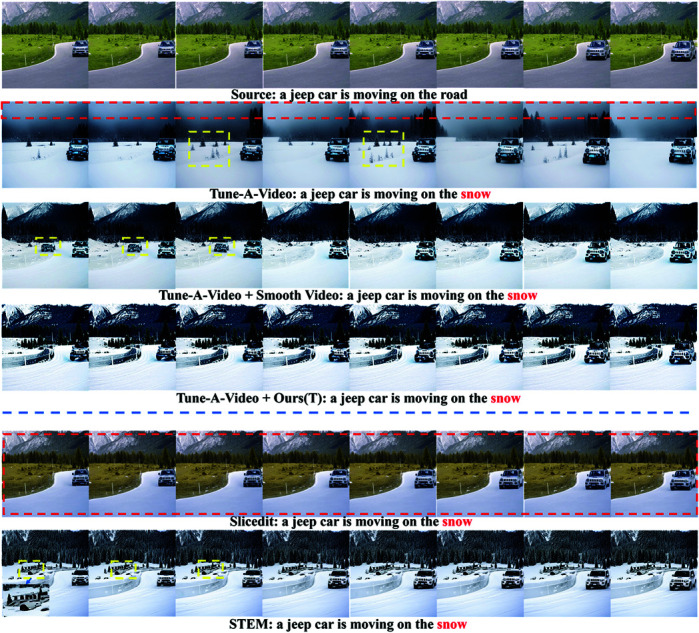
Comparison of smoothing editing results of outdoor scene video generated by Tune-A-Video.

Fig 6 shows the experimental results of the first 8 frames selected from the generated video. As shown by the red box of Tune-A-Video, there are obvious background differences between the frames, resulting in flickering and unsmooth video. Smooth Video improves the smoothness of Tune-A-Video, but there are obvious generation anomalies in the first three frames (as shown in the yellow boxes of Smooth Video). Similarly, Slicedit does not achieve text alignment. The generation results of STEM are abnormal, as shown by the yellow boxes, additional cars that do not exist in the source video appear. Compared with the above methods, the proposed method (Ours(T)) can make the background texture of the generated video clearer and can improve the interframe consistency. Meanwhile the proposed method achieves text alignment, and preserves the temporal consistency with the source video.

**Fig 7:**
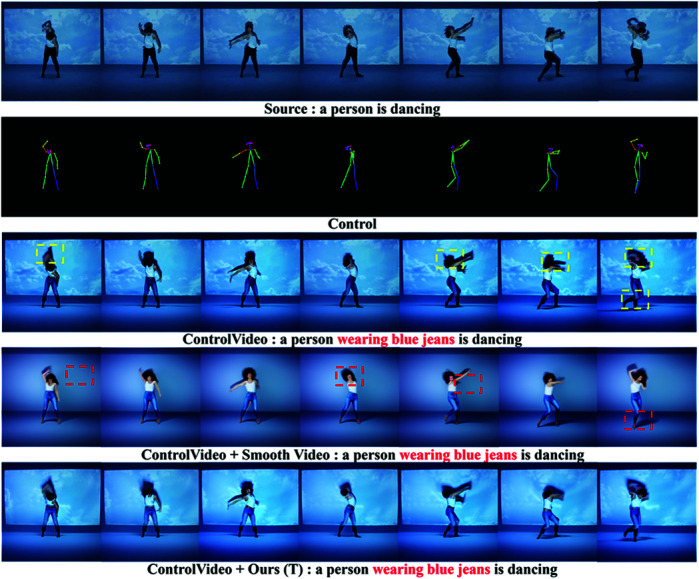
Comparison of smoothing editing results of human pose guided video generated by ControlVideo.

**Comparison with Control Video.** The experimental results of the baseline method ControlVideo [9], the comparison method Smooth Video [13] and Ours(T) are compared. The smoothing loss weight of Ours(T) is set to 0.2. Fig 7 shows the first 7 frames results of the experiments. As the figure shows, while ControlVideo can generate text-aligned videos, there are still shortcomings in the shadows and leg structure of the person in the video (as shown in the yellow boxes). Smooth video, improved character quality in some frames, but due to over-smoothing, the background texture disappears, some action poses are distorted, and cannot be aligned with the source video (as shown in the red boxes). In contrast, the proposed method aligns better with the background, color, and texture of the source video, while improving the person’s shadows and poses, and maintaining temporal consistency with the source video.

### Smoothing results based on training-free baselines

**Comparison with FateZero.** Due to FateZero [23]’s advantages in video content style and structural shape editing, the generated videos have better temporal consistency and smoothness. Under the condition that guidance_scale is set to 7.5, an style transfer experimental comparison is conducted between FateZero, Slicedit [27], STEM [40] and the proposed video smoothing editing method (Ours(TF)) which is based on the training-free models. The inverse noise weight $\lambda_{r}$ of Ours(TF) is set to 1.0.

**Fig 8:**
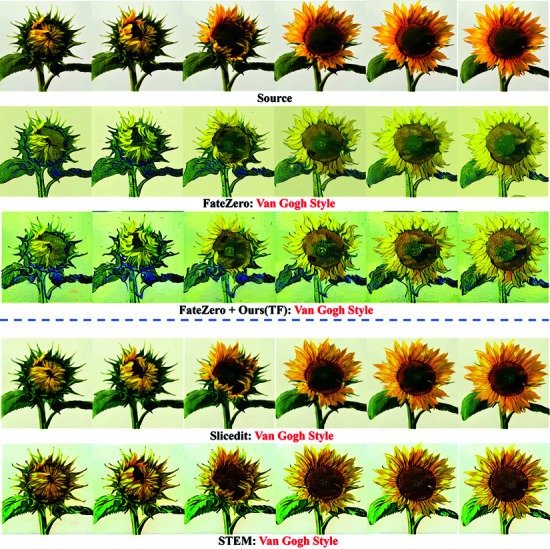
Comparison of smoothing editing results of the stylized video generated by FateZero.

**Fig 9:**
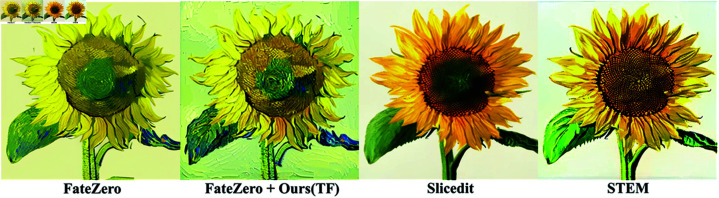
Comparison of video background texture stylization effect.

Fig 8 shows that the first three frames of FateZero have a strong stylized effect, whereas the last three frames have a weak stylized effect, displaying inconsistent stylized effect. The generation effect of Slicedit is close to the source video and does not achieve the Van Gogh style. The artistic effect of STEM needs to be strengthened, and the background does not realize the corresponding artistic style. In terms of style editing, the proposed method (Ours(TF)) demonstrates overall consistency, and the texture artistic effect of leaves and petals in the last three frames is consistent with that of the first three frames, and the background of each frame also shows consistency with the style (as shown in Fig 9). Through comparison, it is found that the proposed method not only maintains the alignment with the text, but also has obvious advantages in terms of overall artistic style, color, and texture presentation.

**Comparison with Video-P2P.** Video-P2P [26] can achieve better application in generating new videos by changing the guide text and attention weighting, and has good performance in maintaining frame consistency. In this paper, Video-P2P is selected to compare with Slicedit [27], STEM [40], and Ours(TF). The inversion noise weight $\lambda_{r}$ of Ours(TF) is set to 0.5.

**Fig 10:**
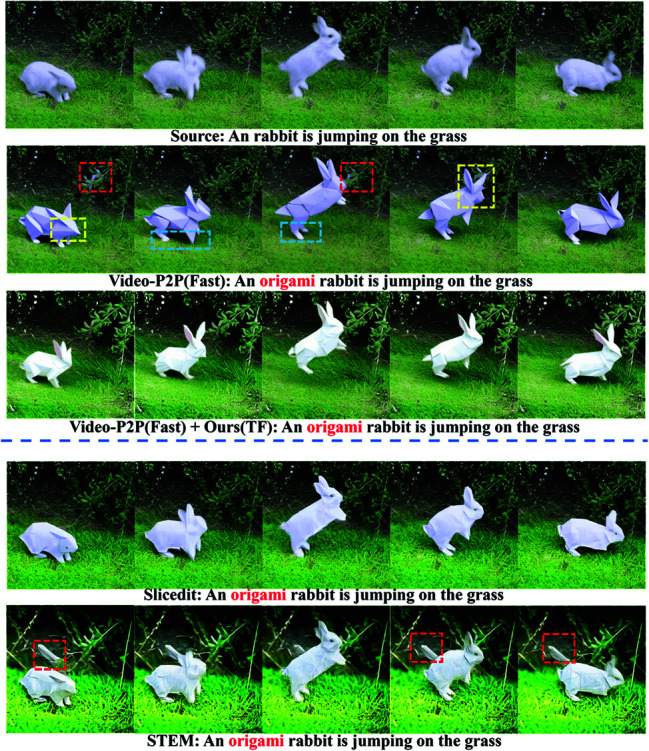
Comparison of smoothing editing results of semantic style video generated by Video-P2P.

As shown in Fig 10, the results of Video-P2P show many inconsistencies, such as inconsistent background and texture (as shown in the red boxes of Video-P2P), inconsistent structure and shape of rabbit head (as shown in the yellow boxes of Video-P2P), and inconsistent color and shape of rabbit feet (as shown in the blue boxes of Video-P2P). Inconsistent backgrounds and textures similarly appear in the STEM results. In contrast, Slicedit and the proposed method Ours(TF) achieve text alignment, however, the origami effect of Slicedit needs to be strengthened.

### Quantitative results

In this section, the training and training-free video smoothing methods proposed in this paper are quantitatively compared with the following T2V baselines, including Tune-A-Video [8], ControlVideo [9], Smooth Video [13], FateZero [23], Video-P2P [26], Slicedit [27], and STEM [40]. Figs 4, 5, and 7 show that the results of Slicedit are almost identical to the source video and are not aligned with the text. Therefore, Slicedit is excluded from these quantitative comparisons. Tables 1 and 2 display the results of the quantitative comparison. CLIP (T) represents the degree of alignment between the generated video and the text, so the higher the value, the better the alignment. CLIP (F) further indicates the frame consistency of the generated video, so higher value indicates the better smoothness. The purpose of optical flow metric is to compute the motion vector of each pixel between two frames using image information from succeeding frames in order to determine the video’s smoothness.

**Table 1:** Quantitative comparison with training baselines.

Method	CLIP (T) *↑*	CLIP (F) *↑*	Optical flow *↓*
Tune-A-Video	0.294	0.960	0.140
Tune-A-Video + Smooth Video	0.258	0.968	0.128
Tune-A-Video + Ours(T)	**0.296**	**0.972**	**0.117**
STEM	0.290	0.968	0.130
ControlVideo	0.249	0.932	0.095
ControlVideo + Smooth Video	**0.277**	0.932	**0.063**
ControlVideo + Ours(T)	0.242	**0.933**	0.094

**Table 2:** Quantitative comparison with training-free baselines.

Method	CLIP (T) *↑*	CLIP (F) *↑*	Optical flow *↓*
FateZero	0.306	0.965	**0.161**
FateZero + Ours(TF)	**0.317**	0.960	0.182
STEM	0.261	**0.988**	0.165
Video-P2P	0.305	0.945	0.142
Video-P2P + Ours(TF)	**0.306**	**0.951**	**0.138**
Slicedit	0.303	0.942	0.141
STEM	0.289	0.948	0.147

As shown in Table 1, through quantitative comparison, the proposed video smoothing editing method (Ours(T)) which is based on the training models, is significantly superior to the compared baselines in the comprehensive scores of the three metrics: text alignment CLIP (T), frame consistency CLIP (F), and Optical flow. In the experiments based on ControlVideo, the shadow of jeans is smoothed out due to the over-smoothing of Smooth Video, so the CLIP (T) and optical flow metrics of Smooth Video are improved, but the experimental results of the proposed method (Ours(T)) are more consistent with the background texture and light shadow of the source video.

Table 2 shows that the proposed video smoothing editing method (Ours(TF)), based on training-free models, significantly improves text alignment, frame consistency, and temporal consistency. It is worth noting that when the proposed method (Ours(TF)) is used to smooth the stylized video generated by FateZero, the art style texture effect improves, resulting in a higher CLIP (T); however, since the background of each frame also implements the artistic style, the CLIP(F) and optical flow metrics of the proposed method are relatively poor compared with the solid color background of FateZero and STEM.

The preceding studies demonstrate that the proposed methods have significant effects in text-to-video editing. We extensively evaluate the training and inference time of the training and training-free methods. For example, Tune-A-Video takes about 12 minutes to train on a 24-frame video with 500 epochs and requires 15.8G of memory resources. Compared with Tune-A-Video, the proposed method (Ours(T)) requires the same training time as Tune-A-Video, and occupies 18.8G of memory resources. It can be seen that the proposed method increases the memory resources by about 128M per frame while editing the training baseline, but does not add extra computation time. The improvement in text alignment, temporal consistency, and fidelity makes the additional memory resources worthwhile. Training-free method FateZero takes about 1 minute 32 seconds to infer 8 frames of video and requires 9.7 to 11.4G of memory resources. Our method (Ours(TF)) takes the same computational cost as FateZero. It can be seen that the computational resources required to edit a training-free model mainly depend on the baseline model. For various text inputs such as scene, dress, style, and material, compared with the state-of-the-art methods, the proposed method can generate consistent video content that conforms to the semantics of the text. For the additional guidance conditions, such as human pose, the proposed method can also achieve alignment with the source video and guide pose. The above stable generation performance further verifies the robustness of the proposed method.

### Ablation study

**Fig 11:**
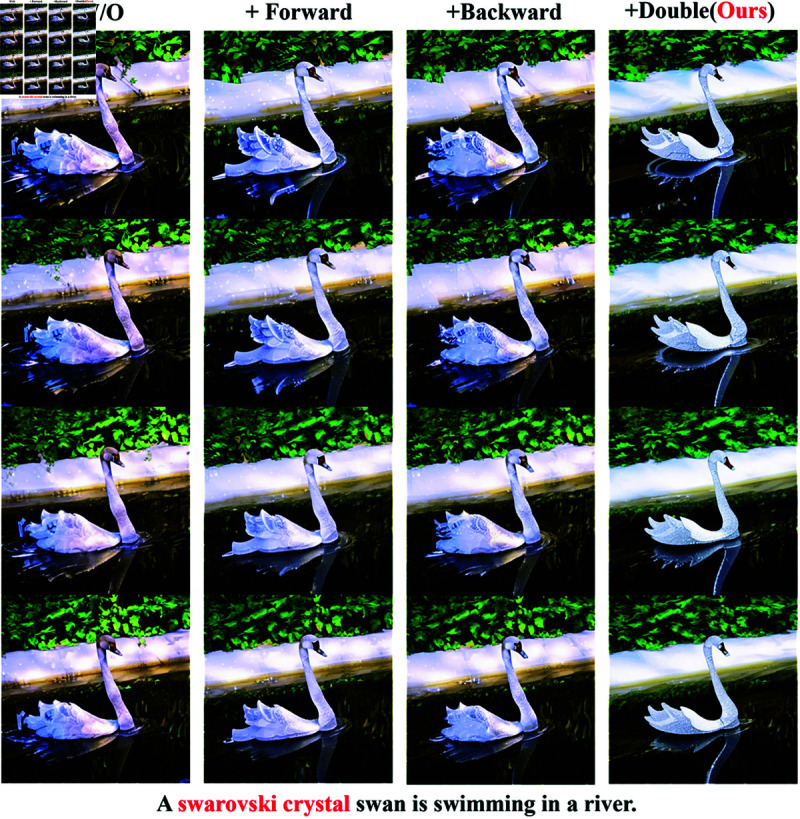
Ablation study. We ablate the following components: (Forward) noise constraint from the last frame to the first, (Backward) noise constraint from the first frame to the last.

We conduct ablation experiments by removing the forward noise constraint (Forward) and the backward noise constraint (Backward) from our frame work, respectively, as shown in Fig 11. Ablation experiments reveal that using the forward noise constraint can improve text alignment and local properties. For example, the material of the swan head and neck is improved, and the structure shape of the swan tail is improved. The use of the backward noise constraint also improves the text alignment, the material of the swan head and neck is improved, but the shape of the swan tail does not change significantly. By adding noise constraints in both directions at the same time (Double), the overall material and structure shape achieve more significant improvement, both text alignment and temporal consistency are enhanced.

## Limitations

Qualitative and quantitative experiments show that the proposed method achieves superior text alignment and source video fidelity, and the generated video is temporal consistent. However, it is worth noting that the temporal consistency of the edited video is affected by the quality of the source video and the generation effect of the baseline method. Since the proposed method edits the generated results of the baseline method, if the generated results of the baseline method fully depart from the text semantics or the content of each frame is extremely diverse, the editing enhancement effect will be obscured.

## Conclusion

This paper proposes a video smoothing strategy INR Smooth, which is based on the interframe noise relation. Based on INR Smooth, two specific video smoothing editing methods are proposed for training models and training-free models. A large number of experiments have proved that the proposed methods have achieved remarkable results in a wide range of applications. The proposed methods have obvious advantages in maintaining the consistency between video frames, text alignment and temporal consistency with the source video, and has outstanding performance in the smooth transition of real scenes, the portrayal of artistic styles, and the maintenance of guiding conditions.
